# Ontogeny of electric organ and electric organ discharge in *Campylomormyrus rhynchophorus* (Teleostei: Mormyridae)

**DOI:** 10.1007/s00359-020-01411-z

**Published:** 2020-02-28

**Authors:** Linh Nguyen, Victor Mamonekene, Marianne Vater, Peter Bartsch, Ralph Tiedemann, Frank Kirschbaum

**Affiliations:** 1 Unit of Evolutionary Biology/Systematic Zoology, Institute of Biochemistry and Biology, University of Potsdam, Karl-Liebknecht-Str. 24-25, Haus 26, 14476 Potsdam-Golm, Germany; 2 Unit of Biology and Ecology of Fishes, Faculty of Life Sciences, Humboldt University of Berlin, Philippstr. 13, Haus 16, 10115 Berlin, Germany; 3 Ecole Nationale Supérieure d’Agronomie et de Foresterie, Université Marien Ngouabi, B.P. 69, Brazzaville, Republic of Congo; 4 Unit of General Zoology, Institute of Biochemistry and Biology, University of Potsdam, Karl-Liebknecht-Str. 24-25, Haus 26, 14476 Potsdam-Golm, Germany; 5 Leibniz-Institute for Evolution and Biodiversity Science, Museum fuer Naturkunde Berlin, Invalidenstr. 43, 10115 Berlin, Germany

**Keywords:** Weakly electric fish, Development, Electric organ discharge, Electric organ, Electrocyte features

## Abstract

The aim of this study was a longitudinal description of the ontogeny of the adult electric organ of *Campylomormyrus rhynchophorus* which produces as adult an electric organ discharge of very long duration (ca. 25 ms). We could indeed show (for the first time in a mormyrid fish) that the electric organ discharge which is first produced early during ontogeny in 33-mm-long juveniles is much shorter in duration and has a different shape than the electric organ discharge in 15-cm-long adults. The change from this juvenile electric organ discharges into the adult electric organ discharge takes at least a year. The increase in electric organ discharge duration could be causally linked to the development of surface evaginations, papillae, at the rostral face of the electrocyte which are recognizable for the first time in 65-mm-long juveniles and are most prominent at the periphery of the electrocyte.

**Electronic supplementary material:**

The online version of this article (10.1007/s00359-020-01411-z) contains supplementary material, which is available to authorized users.

## Introduction

The speciose weakly electric gymnotiform and mormyrid fishes (Froese and Pauly 2008) possess an active sensory system comprising electric organs (EOs) and electroreceptors (e.g., Moller 1995). This system is used for electrolocation (e.g., Bullock and Heiligenberg 1986; von der Emde 1992, 1999, 2006) and electrocommunication (e.g., Kramer 1990).

The electric organ discharges (EODs) are very diverse both in gymnotiform (e.g., Crampton 2006; Crampton et al. 2013) and mormyrid fishes (e.g., Hopkins 1981; Lamanna et al. 2016). Early investigations on the ontogeny of the EOs suffered from a lack of embryological material and juveniles (Keynes 1961; Szabo 1960, 1966). The reproduction of these weakly electric fishes is based on the imitation of rainy season conditions (Kirschbaum 1975) and opened the way for detailed ontogenetic descriptions (Kirschbaum and Schwassmann 2008; Kirschbaum 1977, 1981). This led to the discovery of larval electric organs (Kirschbaum 1977).

African mormyrid fish possess a larval EO found in the deep lateral muscle and an adult electric organ located in the caudal peduncle. The EOD of the larval organ is biphasic, with a large head-positive phase and a smaller, longer lasting head-negative phase; the total duration is ca. 3 ms (Kirschbaum and Westby 1975). The first description of such a larval electric organ was performed on *Pollimyrus isidori* (Westby and Kirschbaum 1977). Similar larval EODs were found in *M. rume proboscirostris* and in *Campylomormyrus cassaicus* (Schugardt 1997), in *P. castelnaui* and *P. marianne* (Baier et al. 2006), in *Marcusenius macrolepidotus* (Werneyer and Kramer 2006), in *Petrocephalus soudanensis* (Kirschbaum, unpublished results), in *Campylomormyrus compressirostris*, *C. tamandua*, *C. tshokwe* and in three morphs of *Paramormyrops* sp. “*magnostipes*” (Nguyen, unpublished results). The larval EOD is not species specific and cannot be used for species identification.

The larval electrocytes are located in the myomeres of the deep lateral muscle and border muscle fibers (Kirschbaum 1977, 1981). They contain many partly disorganized myofibrils and a caudad stalk which receives the innervation (Denizot et al. 1978). The anatomy of the larval electrocytes, first discovered in *P. isidori* (Kirschbaum 1981), was found similar in other mormyrid species, i.e., *Hyperopisus bebe*, *M. rume*, and *Mormyrops deliciosus* (Kirschbaum 1995). Therefore, the larval electric organ of the mormyrids is a very conservative structure. It degenerates early in ontogeny (Kirschbaum 1981) after the differentiation of the adult EO.

The adult electric organ in the caudal peduncle has been repeatedly described (e.g., Koelliker 1849; Schlichter 1906; Szabo 1958; Bennett 1971; Bruns 1971; Bass 1986). It represents a very complex structure which develops in ontogeny later than the larval EO. At a certain stage, both organs coexist and are both active (Westby and Kirschbaum 1978). In *P. isidori* the discharges of the two organs do not superimpose, instead, the activity of the larval organ is separated from the following activity of the adult organ by about 0.6 ms (Westby and Kirschbaum 1978; Baier et al. 2006). Therefore, changes in amplitude of both organs and the disappearance of the activity of the larval organ are easily observable. In contrast, discharges of both electric organs are superimposed during their co-existence in *M. macrolepidotus* (Werneyer and Kramer 2006), *M. rume proboscirostris* and *C. cassaicus* (Schugardt 1997), *P. soudanensis* (Kirschbaum, unpublished results), *C. compressirostris*, *C. tamandua*, *C. tshokwe*, and in three morphs of the *Paramormyrops* sp. “*magnostipes*” (Nguyen, unpublished results), rendering the activities of the two respective organs more difficult to disentangle. This period of co-existence lasts about 40 days in *P. isidori* (Westby and Kirschbaum 1978) and about 3 weeks in *P. castelnaui* and *P. marianne* (Baier et al. 2006).

The complex structure and diversity of the adult EO (e.g., Bass 1986) is matched by a high diversity of EOD waveforms (e.g., Hopkins 1981; Bass 1986; Lamanna et al. 2016). The EOD can serve as prezygotic isolation mechanism and can be used for species identification.

EODs are quite short (in the range of several 100 µs up to about 1 ms) and—once the adult EOD is established during early ontogeny in the juvenile fish—it will not further change during ontogeny up to adulthood. Exceptions from this rule are the Type I and Type III morphs of *Paramormyrops* sp. “*magnostipes*” (Arnegard et al. 2005), where some change in the shape of the EOD occurs during ontogeny. Furthermore, deviating from this pattern is the ontogeny of *Campylomormyrus numenius*, a species with a very long EOD of about 25 ms duration (Paul et al. 2015). Juvenile fish of about 10 cm total length produce an EOD which is considerably shorter and different in shape, relative to that of the adult. A complete description of the EOD ontogeny of this species could so far not be performed due to the lack of captive breeding. The sister species of *C. numenius* is *C. rhynchophorus* (Feulner et al. 2008), which also produces an adult EOD of about 25 ms duration. As this species can be bred quite easily in captivity (Nguyen et al. 2017), we choose this fish for a longitudinal study on the ontogeny of the EOD. We could indeed show, that the adult EOD changes during ontogeny over several months considerably both in shape and duration, a pattern which so far has never been described in any mormyrid fish.

## Materials and methods

### Animals and breeding

The *C. rhynchophorus* pair that gave origin to the developmental stages described in this paper originated from the Congo River from the Kinshasa region. It was purchased via a wholesale dealer in Germany (near Frankfurt) and it grew up from juvenile size up to the size of sexual maturity (ca. 15 cm). Imitation of rainy season conditions consisted of lowering the water conductivity in the experimental tanks. Subsequently the fish developed ripe gonads and spawned repeatedly. Details of the breeding are described in Nguyen et al. (2017).

The parental fish were fed twice daily with live *Chaoborus* (*Corethra*) larvae in the morning and live chironomids in the evening. Frozen food was supplied when live food was not available.

The inseminated eggs were put into Petri dishes (*ø* = 8.6 cm, 1.5 cm high) with various densities between 5 and 50 eggs per dish. At one day postspawning, the eggs were inspected for viable embryos and unfertilized eggs were discarded. Only the fertilized eggs were selected for further incubation in the water of the breeding tank. Incubation was performed in an incubator at 27 ± 0.5 °C or at room temperature (23–27 °C). After hatching, free embryos were transferred into new Petri dishes and tap water (conductivity ca. 700 µS/cm; pH ca. 7.4) was added gradually to the water of the breeding tank to rear them. One week after hatching, the free embryos [(terminology after Balon (1975)] could be raised in tap water. 15-mm-long larvae up to 40-mm-long juveniles were raised in plastic boxes (1 L). Afterwards they were moved to small aquaria (12 L).

The fry were fed with newly hatched *Artemia* nauplii from the beginning of exogenous feeding (i.e., at day eight after hatching) up to about 30- to 40-mm-long juveniles. Thereafter, small chironomid larvae were supplied in addition. Larger juveniles were fed as the parents.

Five specimens were raised individually after the first EOD measurement to follow their ontogeny with regard to both morphology and EOD (after the beginning of exogenous feeding). Additional fish were raised in community tanks and they were checked regularly for growth and EOD development and compared to the individually raised ones. A few of these additional fish were killed for histological investigation of the EO: 18-mm-long fish for the characterization of the larval electric organ; juveniles between 32 and 36 mm long at the transition from the larval to the juvenile EOD; juveniles between 65 and 80 mm length characterized by an EOD of increased duration (ca. 9–37 ms duration); adult fish with an EOD duration of about 25 ms.

### Photography

The specimens used for the morphological description were anesthetized with MS 222. Recovery from the anesthetic took a few minutes. Photos were taken with a digital camera Canon EOS 350D or Canon EOS 100D.

### Oscilloscope recordings

In order to analyze species-specific EOD characteristics, the fish were put in plastic aquaria of different size filled with water of the keeping tank (in general tap water of conductivity values between 700 and 900 µS/cm, temperature: 24–26 °C); the sizes of the recording tank comprised 11 × 11 mm for the larvae, 7 × 5 cm for the smaller, and 13 × 15 cm for the larger juveniles. Water level was adjusted according to the size of the fish. The EOD of the 8-day-old larva was measured in a plastic pipette. The EOD was also recorded in a clay tube in order evaluate whether the plastic aquaria had some influence on the shape of the EOD, which was not the case. The container featured a compartment adjustable in size that prevented the fish from freely swimming during the measurements. Steel electrodes were positioned at the rostral and caudal end of the fish—the positive electrode was positioned next to the head. The EOD waveforms were displayed and recorded by a Tektronix TDS 3012B digital phosphor oscilloscope (sampling rate: 10 kHz; 9 bit vertical resolution) with a Tektronix ADA 400A differential preamplifier (variable gain from 0.1× up to 100×; bandwidth 100 Hz–1 MHz). The oscilloscope and preamplifier were AC coupled. The EOD data were transferred onto the hard drive of a computer via floppy disks. Per individual, one representative EOD, normalized to 1 V, was analyzed.

**Fig. 1:**
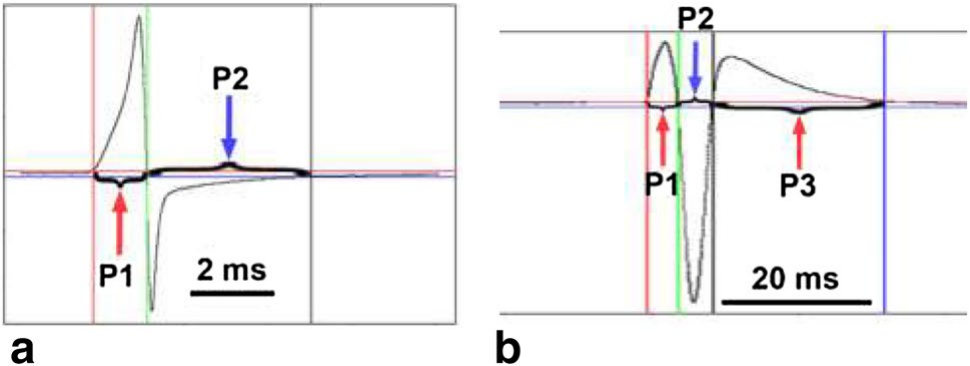
Two electric organ discharge types (EODs) of *Campylomormyrus rhynchophorus* which occur during ontogeny, a biphasic EOD (**a**, from a 65-mm-long juvenile) and a triphasic EOD from a 155-mm-long juvenile (**b**), are selected to illustrate how the duration of the different EOD phases were calculated

The start and end points of whole EODs were defined as the points where the amplitudes reached 2% of the total discharge amplitude. The start and endpoints of each phase within the EOD were determined at the crossing points at the zero line (Fig. 1). Software running under R (see supplementary material) was used to calculate duration and amplitude of each phase and the whole signal.

### Histological techniques

The EOs were subjected to anatomical examinations via light microscopy. For the preparation of the histological slides, the fish were euthanized by an overdose of ethylenglycolmonophenylether and the caudal peduncle was cut off. The tissue was fixed by immersion in 4% formalin in the case of small fish or in 10% formalin in larger fish. Fixed tissue of larger fish was decalcified with 7.5% EDTA (pH 7.4). Dehydration and embedding in paraffin followed established protocols (Mulisch and Welsch 2010). The EO of each specimen was sectioned serially at 4–6 µm either in the transverse or the sagittal plane using a Leica 2035 Biocut microtome. Sections were mounted on glycerin/albumin-coated slides. Following deparaffinization and rehydration, sections were stained with Azan or HE (Mulisch and Welsch 2010), dehydrated and coverslipped with DePeX mounting medium (Sigma-Aldrich). Sections were investigated with a Leica microscope DM4000B. Pictures were taken and processed by using the Leica DFC 480 camera together with the Leica IM software version 4.0.

## Results

### Ontogeny of morphology and of the electric organ discharge

**Fig. 2:**
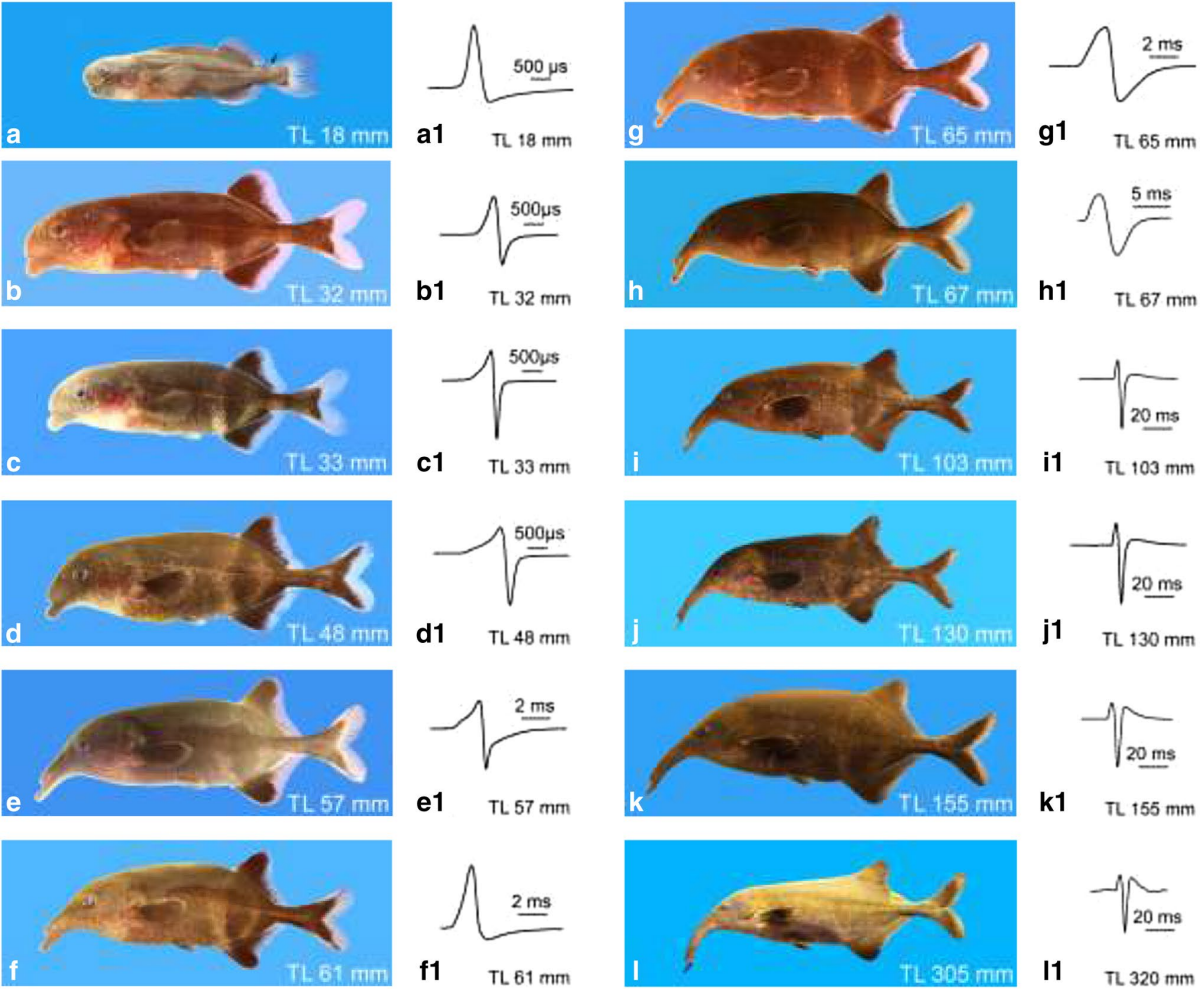
Ontogeny of morphology and electric organ discharge (EOD) of *Campylomormyrus rhynchophorus.* **a** A 18-mm-long larva with remnants of the larval fin fold at the caudal peduncle (arrow). **b**–**k** Juveniles of various sizes showing the change in pigmentation, the allometric growth of the snout and the increase in body height during development. **l** Adult female. **a1** Shows the larval EOD, **b1** the transitional stage with both, the larval and the adult EO active. **c1** EOD of the adult EO alone; we termed this EOD the juvenile EOD, because it is produced by a juvenile fish. Note that the EODs are shown at different time scales to better demonstrate the shape of the EOD. Some data on the morphological and EOD development had already been published in Nguyen et al. (2017)

The first EOD activity was detected in 8-day-old, ca. 10-mm-long free embryos (EOD data not shown here). This biphasic EOD resembles the activity of the larval electric organ already described in other mormyrid species, e.g., in *Pollimyrus* species (Westby and Kirschbaum 1978). The larval EOD of a 18-mm-long larva is shown in Fig. 2a1. This larva still possesses remnants of the embryological fin fold (Fig. 2a); the *Campylomormyrus*-typical snout is not yet developed. A 32-mm-long juvenile (Fig. 2b) produced an EOD (Fig. 2b1) with a first change in the EOD shape: the amplitude of the head-negative phase has started to increase. This is the first indication that the adult electric organ has started to become functional. A 33-mm-long juvenile (Fig. 2c) produced a biphasic EOD where the amplitude of the head-negative phase was about twice as high as that of the head-positive phase (Table 1, Fig. 2c1). We have termed this EOD the “juvenile EOD” because it is produced by the juvenile fish and is different from the EOD found in adults. This EOD type persists for several weeks. At around 48 mm length, the duration of the juvenile EOD had slightly increased and the amplitude of the head-negative phase decreased compared to the amplitude of the head-positive phase (Table 1). Such an EOD of a 48 mm juvenile is shown in Fig. 2d1. The morphology of such a juvenile (Fig. 2d) shows that the *Campylomormyrus*-typical snout has started to develop. A 57-mm-long juvenile (Fig. 1e) showed an EOD with increased duration and a prolongation of the head-negative phase (Fig. 2e1; Table 1). Between the 57 mm juvenile and the 61 mm juvenile a considerable change in EOD shape occurs. The 61-mm-long fish produces an EOD with a large head-positive amplitude and a small head-negative phase of long duration (Fig. 2f1) (Table 1). This EOD shape generated by the adult electric organ resembles very much the shape of the larval EOD (Fig. 2a1), though the duration is much longer (nearly four times as long, Table 1). In a slightly longer juvenile (65 mm, Fig. 2g) the head-positive and head-negative phases are nearly of equal amplitude (Fig. 2g1; Table 1). This EOD type is still found in the 67 mm juvenile (Fig. 1h1), but the duration has slightly increased (Fig. 2h1). A second head-positive phase has started to appear in some specimens of this size. The amplitude of this head-positive phase continuously increased with size (Fig. 2i1, j1, k1) as seen in the 103, 130 and 155-mm-long juveniles (Fig. 1i–k; Table 1). Morphologically the 130 and 155-mm-long juveniles already resemble very much the adult fish (Fig. 1l).

**Table 1:** Change of amplitude (Amp) and duration (Dur) during the electric organ discharge (EOD) ontogeny in *Campylomormyrus rhynchophorus*

Size of fish	Amp P1 (v)	Amp P2 (v)	Amp P3 (v)	Amp P1/P2	Amp P3/P1	Dur P1 (ms)	Dur P2 (ms)	Dur P3 (ms)	Total dur (ms)
33 mm	2.12	3.96	–	0.54	–	0.75	0.25	–	1.00
48 mm	1.22	2.34	–	0.52	–	0.98	0.91	–	1.89
57 mm	1.15	1.59	–	0.72	–	2.05	2.82	–	4.87
61 mm	4.30	0.78	–	5.51	–	1.65	6.21	–	7.86
65 mm	1.31	1.21	–	1.08	–	1.93	4.90	–	6.83
67 mm	0.85	1.18	0.09	0.72	0.11	2.88	4.26	29.46	36.60
103 mm	0.87	2.31	0.23	0.38	0.26	3.81	4.84	32.36	41.01
130 mm	0.36	0.78	0.14	0.46	0.39	4.40	4.70	25.35	34.45
155 mm	0.72	2.26	0.53	0.32	0.74	4.71	4.96	21.61	31.28
320 mm	0.22	0.61	0.18	0.36	0.81	3.60	4.78	18.13	26.51

For the different phases of the EOD, see Fig. 1

**Fig. 3:**
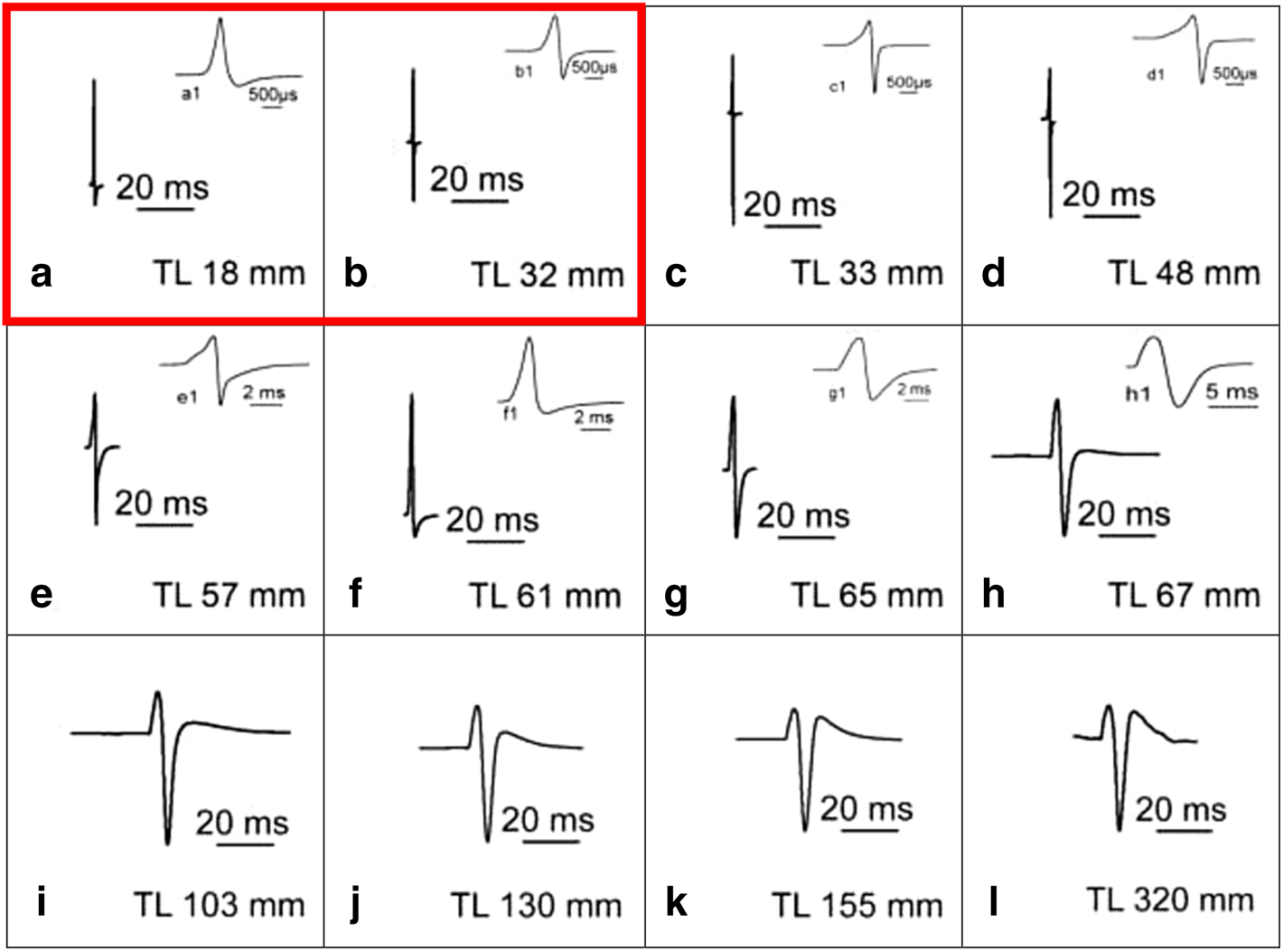
Examples of electric organ discharge (EOD) types observed during ontogeny of *Campylomormyrus rhynchophorus* shown always at two different time scales to demonstrate (1) the change in duration of the EOD (20 ms time scale) and (2) to better show the shape of the EOD and its change during ontogeny (variable time scales, upper EOD). **a** Biphasic larval EOD. **b** Transition from larval to adult EOD; the activities of both EOs are superimposed. **c** First exclusive activity of the adult EO; termed juvenile EOD as it is produced by the juvenile fish. **d**, **e** Change of the juvenile EOD due to an increase in the duration of both phases. **f** Considerable decrease in the amplitude of the head-negative phase; this EOD shape resembles very much that of the larval EOD shown in **a**. **g**, **h** Change into a biphasic EOD of longer duration than before. **i** A triphasic EOD has developed due to the occurrence of a second head-positive phase. The amplitude of this phase continuously increases with the growth of the fish (**j**, **k**, **l**). *TL* total length. Some data on the EOD ontogeny had already been published in Nguyen et al. (2017)

In Fig. 3 the ontogeny of the EOD is shown at two different time bases, (1) with a fixed time scale in order to better illustrate the increase in duration (time scale 20 ms; Fig. 3c–l) and (2) at variable time scale to facilitate EOD shape comparisons (Fig. 3c1–l1). Figure 3a, a1 shows the larval EOD and Fig. 3b, 3b1 the transition between larval and adult EOD.

### Individual variation in the ontogeny of the electric organ discharge

**Fig. 4:**
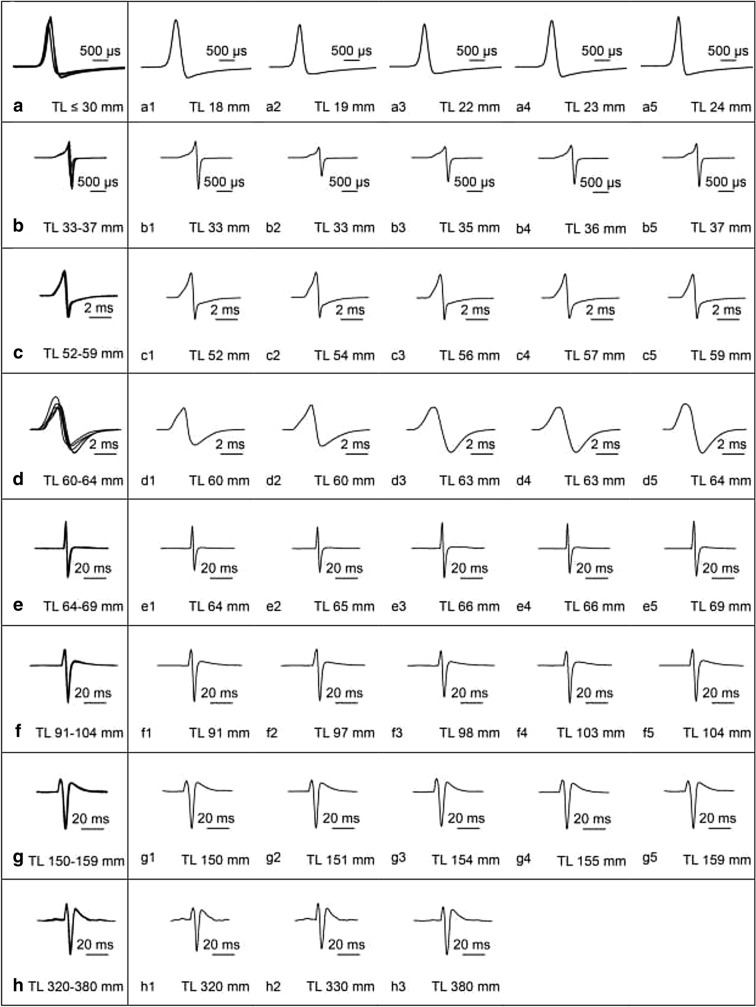
Overlay plots of electric organ discharges (EODs) recorded from five, respectively, three different specimens (**h**) grouped together according to total length and developmental stage in *Campylomormyrus rhynchophorus*. In addition to the overlay plots the individual EODs are shown as well. **a**, **b**, and **d** show slight individual variations in EOD shape, whereas in **c**, **e**–**h** virtually no individual variations in the shape of the EOD did occur. Some data on the EOD ontogeny had already been published in Nguyen et al. (2017)

As shown in the preceding paragraph, the EOD of the adult electric organ changes continuously during ontogeny in both shape and duration, starting from the juvenile EOD in the 33 mm fish (Fig. 3c). Figure 4 shows overlays of EODs (Fig. 4a–h) from five individuals of the same developmental stage (except for the adult EOD, where only 3 specimens were measured) along the EOD ontogeny. The EODs of the individual fish are also shown (Fig. 4a1–h1) in order to illustrate the small difference between EODs of the individuals. Figure 4 starts with the larval EOD (Fig. 4a). Figure 4b shows the earliest juvenile EOD. The EODs of the 52- to 59-mm-long juveniles are shown in Fig. 4c, c1. Biphasic EODs with approximately the same amplitudes of both phases are shown in Fig. 4d, d1, e, e1. Figure 4f, f1, g, g1 shows the EOD of juveniles of which the EOD resembles already very much the EOD of the adult fish (Fig. 4h, h1). Figure 4 demonstrates that there are only slight inter-individual differences in the shape of the EOD during ontogeny; inter-individual differences are most pronounced in 60- to 64-mm-long fish (Fig. 4 d, d1). Among specimens, a specific EOD type does not occur at exactly the same size, but rather within a size range of several mm (Fig. 4).

### Change in the duration of the different phases of the EOD during ontogeny

**Fig. 5:**
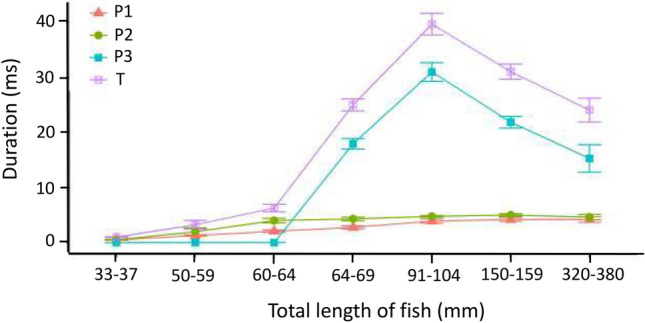
Duration of the different phases of the adult electric organ discharge (EOD) during ontogeny in *Campylomormyrus rhynchophorus.* The large increase in the overall duration of the EOD is brought about by the increase in the duration of the second head-positive phase; the total duration of this phase decreases once the juveniles have reached a size of about 10 cm. Concerning the different EOD phases, see Fig. 1. Mean and standard deviation are shown

As seen in the description of the ontogeny of the EOD (Fig. 2), the originally biphasic discharge changes both its shape and duration and finally develops into a triphasic EOD. Figure 5 shows which of the phases of the EOD contribute to the total duration of the EOD. The duration of the head-positive and the head-negative phases continuously increase nearly in parallel by a factor of about ten up to the largest juveniles. The duration of the head-positive phase (P1) increases from 0.49 ms (33- to 37-mm-long fish) up to 4.47 ms in the 150–159 mm long juveniles (Table 1). The duration of the head-negative phase (P2) increases from 0.50 ms up to 5.01 ms in fish of the same size range. The large increase in the overall duration of the EOD is brought about by the increase in the second head-positive phase (P3); the total duration of this phase decreases once the juveniles have reached a size of about 10 cm (Table 1).

### Histology of the electric organs

#### Larval electric organ

**Fig. 6:**
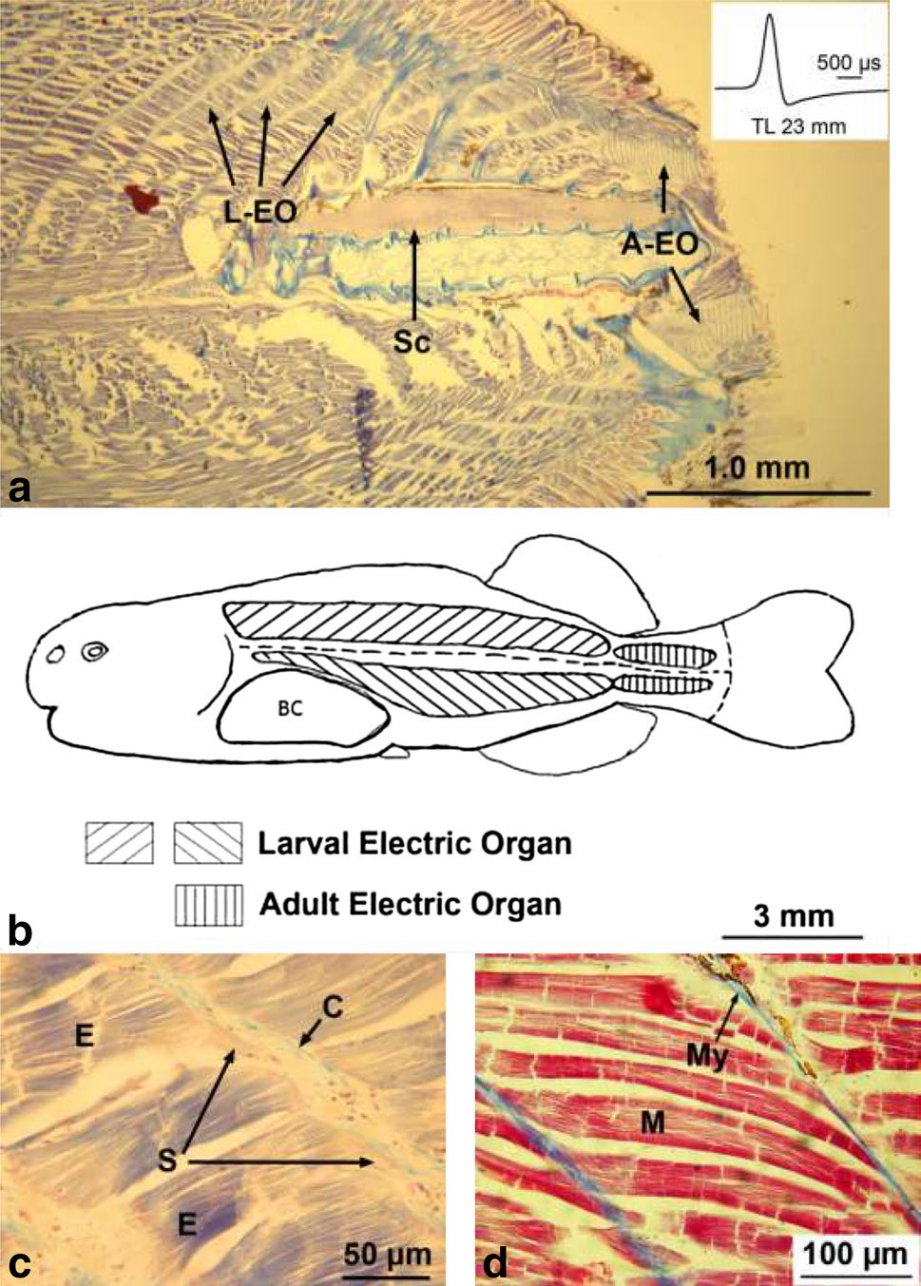
Some characteristics of both, larval and adult electric organ in a 23-mm-long larva of *Campylomormyrus rhynchophorus*. **a** Medial sagittal section showing the position of both electric organs (L-EO, larval electric organ; A-EO, adult electric organ). Note that most of the caudal peduncle has been cut off and that at this stage only the L-EO is active (see inset). **b** Schema of the extension of the L-EO in the deep lateral muscle and of the A-EO in the caudal peduncle. **c** Photomicrograph of a Azan-stained sagittal section showing a few electrocytes (E) of the larval electric organ with their caudad stalk (S) extending into the space filled with loose connective tissue (C). Note the striation of the myofibrils. **d** Photomicrograph of a Azan-stained sagittal section showing a few muscle fibers (M) attached to the myosepts (My)

The electrocytes of the larval EO are found in the deep lateral muscle extending from behind the skull until the beginning of the caudal peduncle (Fig. 6a, b). They are inserted into the myomeres in between the lateral muscle fibers (Fig. 6a) as described in *P. isidori* (Kirschbaum 1981). The myosepts disappear at the level of the larval EO and are replaced by a mass of loose connective tissue (Fig. 6c). The larval electrocytes (Fig. 6c) are thicker than the neighboring muscle fibers (Fig. 6d) and—in contrast to the more densely stained muscle fibers—they possess a caudal extension, a stalk receiving the innervation (Fig. 6c).

#### Adult electric organ

**Fig. 7:**
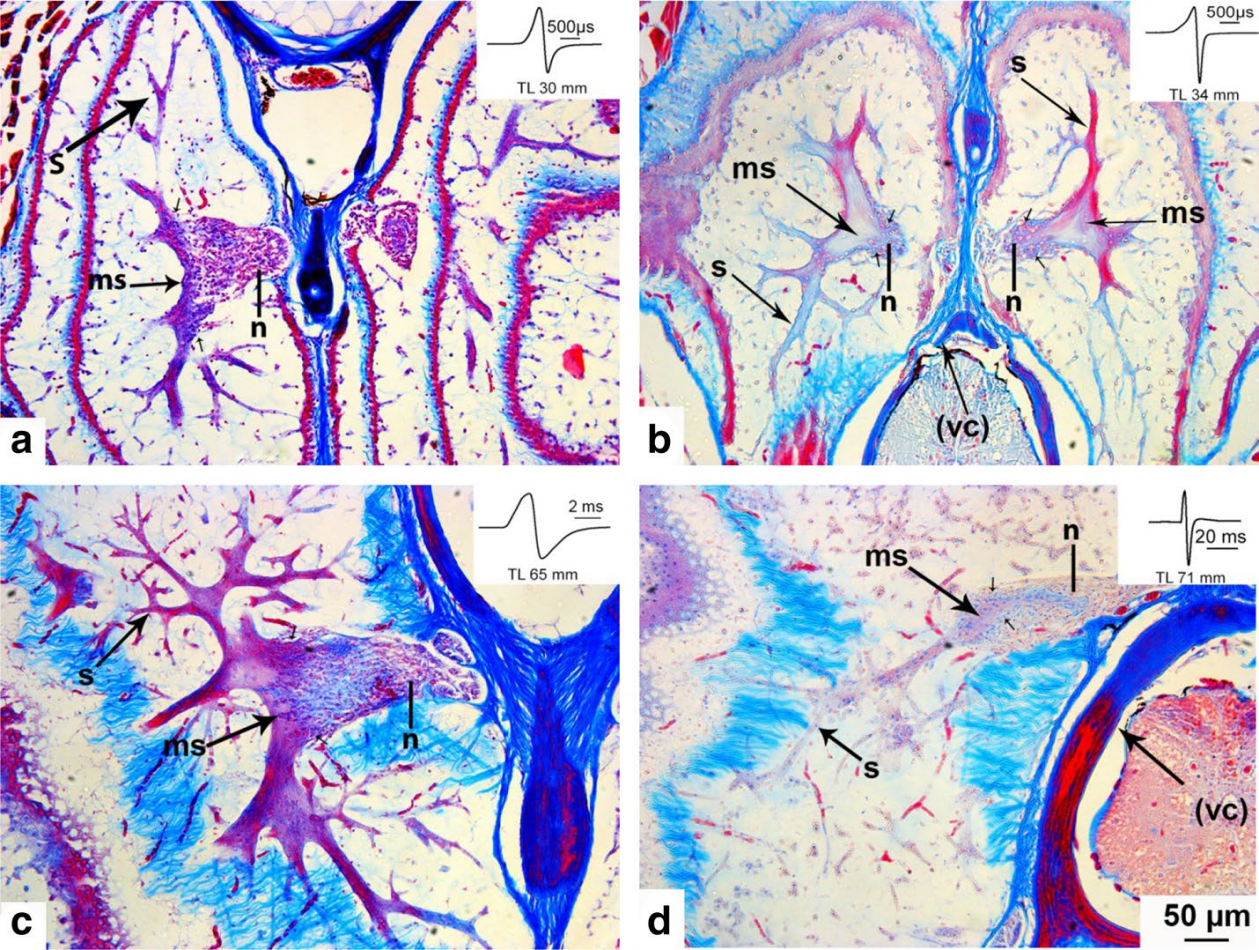
Photomicrographs of Azan-stained cross sections of the adult electric organ (EO) of four developmental stages (30, 34, 65, and 71 mm long) in *Campylomormyrus rhynchophorus*. Nerve fibers (n) from electromotoneurons situated in the spinal cord extend into the column of electrocytes and slightly bifurcate in both dorsal and ventral direction. The stalk system (s) is highly branched comprising one main stalk (ms) from which many branches (s) originate. **a** In this cross section the main stalk (ms) is covered by nerve fibers (n). **b** The central main stalk (ms) is clearly seen in this section. **c** The bifurcation of the main stalk dorsally and ventrally is particularly well seen in this section. **d** Note the very fine ramifications of the peripheral stalk system. vc, vertebral column

**Fig. 8:**
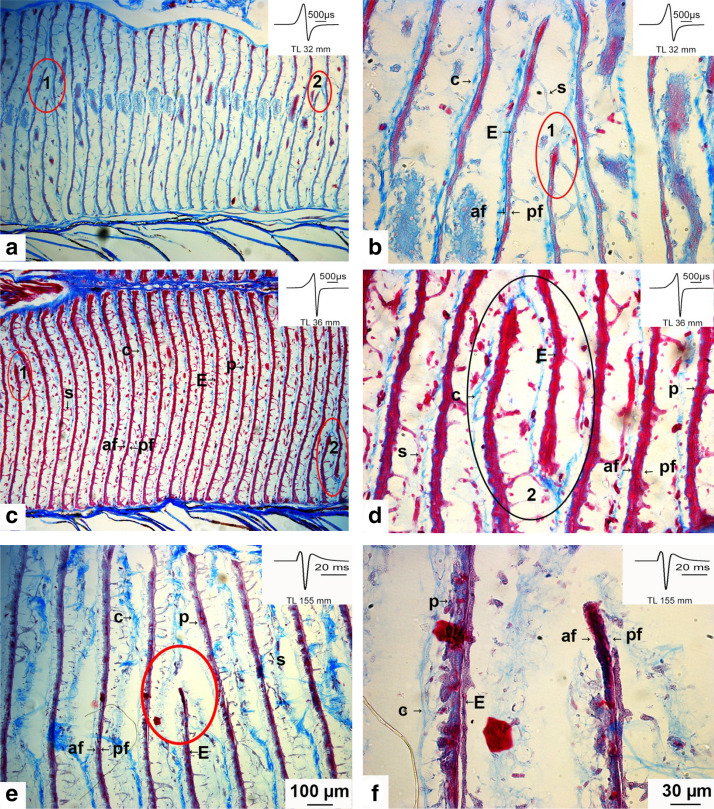
Photomicrographs of Azan-stained sagittal sections showing irregular geometry of some electrocytes in three stages (32, 36, and 155-mm-long juveniles) of *Campylomormyrus rhynchophorus*. These electrocytes are attached either at the dorsal or the ventral face (**a**, **c**) of the electrocyte column but they do extend over the whole width of the column (**b**, **d**, **e**, **f**). *E* electrocyte, *s* stalk, *af* anterior face, *pf* posterior face, *c* connective tissue, *p* papillae

The adult electric organ is situated in the caudal peduncle starting at the level of the caudal end of the dorsal fin and terminates before the beginning of the caudal fin (Fig. 6b). It is composed of four columns of in parallel arranged electrocytes, two ventral and two dorsal ones. Each electrocyte is enclosed in a compartment of loose connective tissue. As analyzed in serial sagittal sections, the main stalk system of the electrocytes of the adult organ in *C. rhynchophorus* is situated on the caudal face of the electrocyte (Fig. 8a). Figure 7 shows the branching type in *C. rhynchophorus* at various ontogenetic stages in cross sections of the EO. The main stalk of a 30 mm larva at the beginning of the activity of the adult EO is seen in Fig. 7a. One branch of the stalk extends dorsally and another one ventrally. From these main branches, additional big branches extend laterally in both directions. In the 34 mm juvenile (Fig. 7b), a similar branching pattern is observed. In addition, it can be seen in this figure that the main stalk approaches the median line and neural tube of the fish; thus the electric nerves innervating the main stalk do not reach very far to the electrocyte. In the 65 mm juvenile (Fig. 7c), the branching pattern of the main stalk is more distinctive than in the younger fish. In the 71 mm juvenile, the finer branching pattern of the stalk system becomes apparent (Fig. 7d) (Table 2).

**Table 2:** Change of the different phases (P1–P3, see Fig. 5) of the electric organ discharge during ontogeny in *Campylomormyrus rhynchophorus*

Size of fish	P1 duration (ms)	P2 duration (ms)	P3 duration (ms)	Total duration (ms)
33–37 mm TL	0.47–0.51 (**0.49**)	0.41–0.56 (**0.50**)		0.92–1.07 (**0.99**)
50–59 mm TL	1.08–1.41 (**1.26**)	1.11–2.72 (**1.99**)		2.71–4.09 (**3.25**)
60–64 mm TL	1.79–2.41 (**2.07**)	3.44–4.59 (**3.99**)		5.47–7.11 (**6.26**)
64–69 mm TL	2.35–3.17 (**2.76**)	3.93–4.75 (**4.29**)	17.08–19.51 (**17.93**)	23.40–26.90 (**24.99**)
91–104 mm TL	3.67–4.69 (**3.92**)	4.53–4.86 (**4.75**)	28.65–32.36 (**30.95**)	37.24–41.68 (**39.62**)
150–159 mm TL	4.22–4.75 (**4.47**)	4.71–5.32 (**5.01**)	20.81–23.19 (**21.84**)	29.87–32.62 (**31.05**)
320–380 mm TL	3.60–4.55 (**4.17**)	4.07–4.97 (**4.61**)	13.73–18.13 (**15.26**)	22.53–26.51 (**24.04**)

Mean values are in bold

The four columns of the adult EO contain a variable number of electrocytes in mormyrids (see e.g., Paul et al. 2015). In *C. rhynchophorus* the number of electrocytes varies between 42 and 52 per column (Table 3). The table also shows that there is no significant increase in the number of electrocytes during early ontogeny.

**Table 3:** Number of electrocytes in the four columns of the electric organ in four stages of juvenile and early adult *Campylomormyrus rhynchophorus*

Size of fish	No. of E in DR column	No. of E in DL column	No. of E in VR column	No. of E in VL column
32 mm	48	50	48	47
36 mm	49	48	42	46
65 mm	46	46	44	47
80 mm	44	46	42	–
155 mm	51	52	51	50

*E* electrocyte, *DR* dorsal right; *DL* dorsal left, *VR* ventral right, *VL* ventral left

The electrocytes extend from the dorsal to the ventral part of the column (Fig. 7a, c, d). However, there are a few irregularities in the alignment of the electric cells: some electrocytes do not extend over the whole width of the column, as demonstrated in Fig. 8b, d, f.

**Fig. 9:**
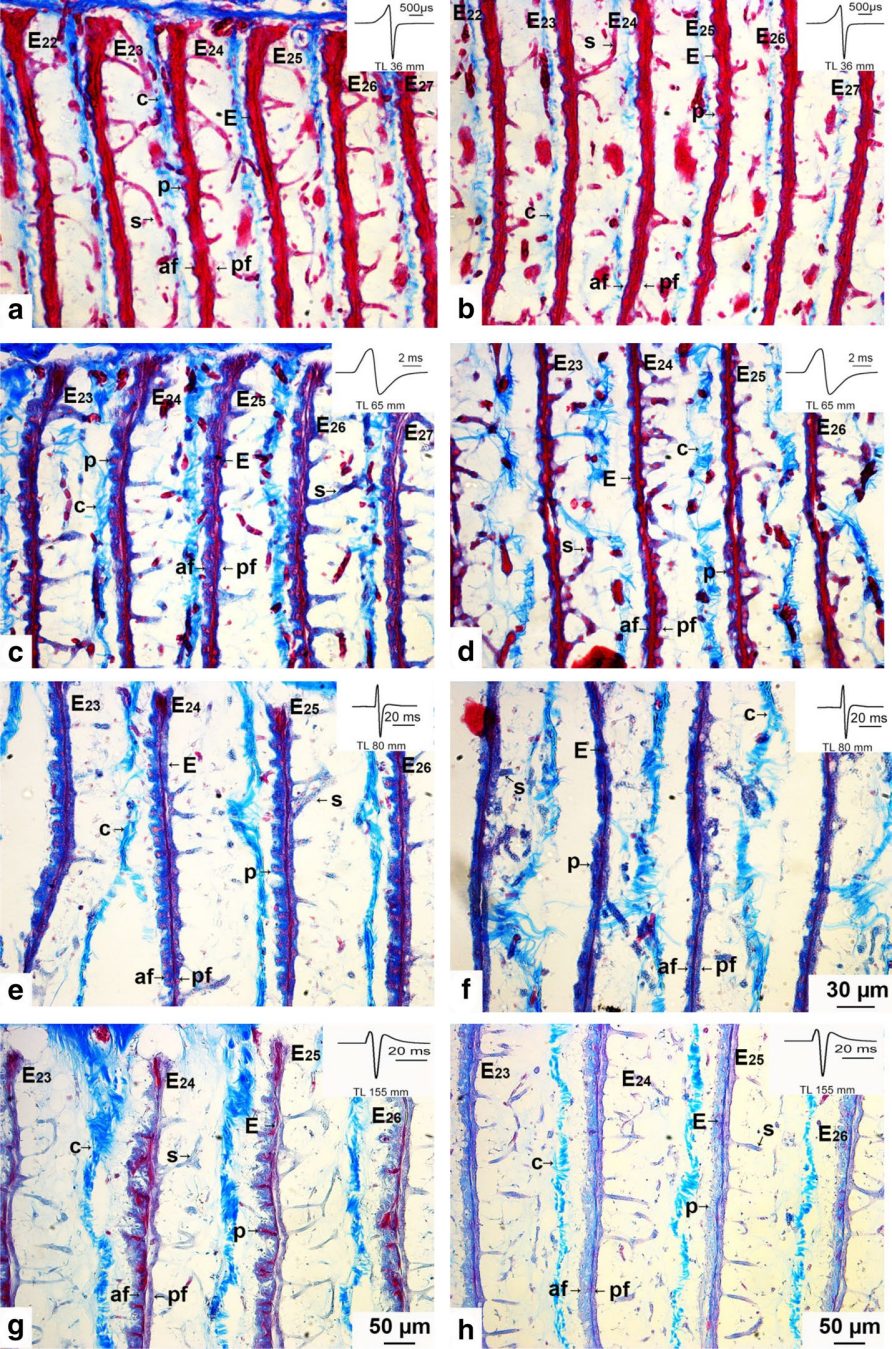
Photomicrographs of Azan-stained sagittal sections of some electrocytes (E) to show papillae-like structures in four stages (36, 65, 80, and 155-mm-long juveniles) of *Campylomormyrus rhynchophorus*. The four pictures in the left column show the periphery of the E, whereas those in the right column are taken from the center of the E. The papillae are more pronounced at the periphery (**a**, **c**, **e**, **g**) than in the center (**b**, **d**, **f**, **h**) of the E. *af* anterior face, *C* connective tissue, *E* electrocyte, *p* papillae, *pf* posterior face, *S* stalk

Surface evaginations of the electrocytes, so-called papillae, were described in many species (Bennett 1970, 1971; Schwartz et al. 1975; Bass et al. 1986). They are thought to contribute to an elongation of the EOD. We also found papillae in *C. rhynchophorus*. Figure 9 shows micrographs of electrocytes from the central part of the EO at various developmental stages. For each stage, peripheral and central regions of the electrocyte are compared. In the 36 mm juvenile (i.e., at the time when the adult EO begins to function), very small surface evaginations are seen both at the periphery (Fig. 9a) and the central part of the electrocytes (Fig. 9b), which seem to comprise structures later developing into papillae. Distinct papillae are found in the 65 mm juvenile, however, these are more pronounced at the periphery (Fig. 9c) than in the central part of the electrocyte (Fig. 9d). In a juvenile of 80 mm length, these papillae are even more densely packed at the periphery (Fig. 9e) than in the younger juvenile (Fig. 9c); again the central part of the electrocyte shows less developed papillae. The 155 mm juvenile also shows less developed papillae in the central part of the electrocyte (Fig. 9h), as compared to the periphery (Fig. 9g). At this stage, the peripheral papillae have a different form, extend even longer from the central axis of the electrocyte and contain a violet-stained center which seems to be composed of myofibrils. These run, similarly stained, through the whole center of the electrocyte, which is a typical feature of the mormyrid electrocyte (see, e.g., Bruns 1971).

**Fig. 10:**
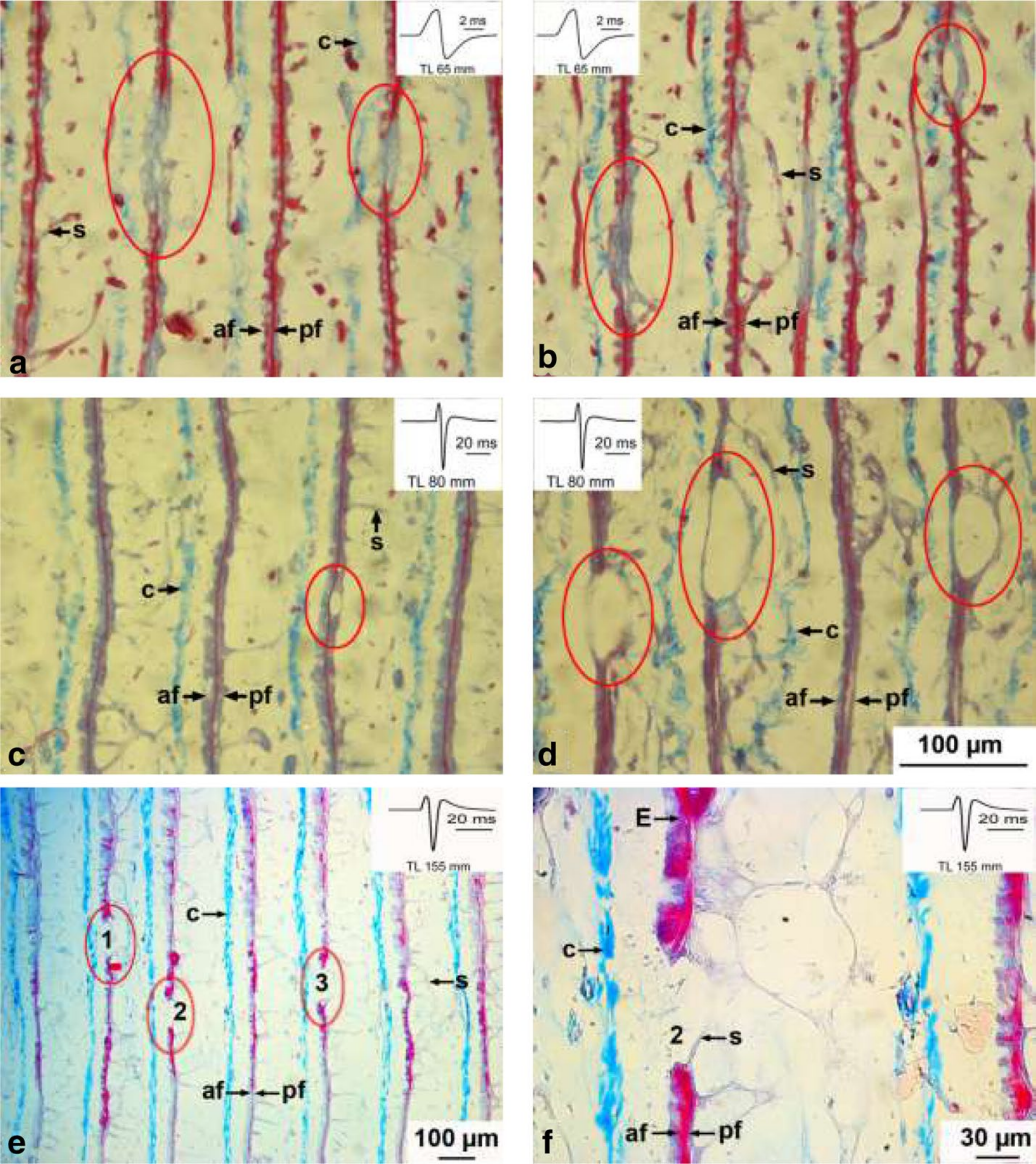
Photomicrographs of Azan-stained sagittal sections of electrocytes (E) exhibiting holes and cavities in three developmental stages (65, 80, and 155 mm long) of *Campylomormyrus rhynchophorus*. **a**, **b** Red oval encompasses an electrocyte (E) portion where the central myofilament bundle is interrupted or where a small cavity has developed (**b**). **c**, **d** Red ovals indicate locations, where cavities have developed. **e** Red oval encompasses E with a complete separation of upper and lower portion of the E (hole). **f** Hole number 2 (see **e**) at higher magnification. Note the alignment of the connective tissue along the E. *af* anterior face, *c* connective tissue, *E* electrocyte, *pf* posterior face, *s* stalk

There are some structural features of the electrocytes of *C. rhynchophorus* which are atypical for electrocytes of mormyrid fish. Some electrocytes contain holes, short interruptions of the cell body, which are less numerous in the small fish, but seem to increase in number during ontogeny (Fig. 10a, b), compared to the largest individual (Fig. 10g, h). First in the 65 mm juvenile, we observed electrocytes in which the central element of the electrocyte widens, thus separating the rostral from the caudal face (Fig. 10c–f). Thereby a kind of cavity develops. Interestingly, the central band of myofibrils often terminates both at the dorsal or ventral face of that cavity (Fig. 10d, f). Whether these cavities later develop into holes, i.e., complete perforations of the cell, is not clear.

In the 15.5 cm fish we have based on the serial sagittal sections performed a first analysis concerning the distribution and the shape of the wholes in the center of the left dorsal column of electrocytes (52 electrocytes, see Table 3): the electrocytes in their dorso-ventral extension measure ca. 4 mm. The wholes in different electrocytes are randomly distributed over the whole length of the 4 mm. The maximum opening of the wholes in the dorso-ventral extension is about 500 µm; their width in the lateral extension is about 100–250 µm.

## Discussion

The aim of this study was a longitudinal description of the ontogeny of the adult electric organ of *C. rhynchophorus* which produces as adult an EOD of very long duration (ca. 25 ms). We could indeed show (for the first time in a mormyrid fish) that the EOD which is produced early during ontogeny in juvenile fish is much shorter in duration and has a different shape than the adult EOD. The change from this juvenile EOD into the adult EOD is a long lasting process of at least a year. Some explanation for the increase in EOD duration could be the development of surface evaginations, papillae, at the rostral face of the electrocyte during ontogeny. We will discuss these findings based on the assumption that both anatomical and physiological changes contribute to these ontogenetic EOD changes.

### Larval electric organ

The EOD of the larval electric organ of *C. rhynchophorus* is similar to that described in other mormyrid species, e.g., in the *Pollimyrus* species (Westby and Kirschbaum 1977; Baier et al. 2006) and in *M. macrolepidotus* (Werneyer and Kramer 2006). Also the anatomy of the larval electrocytes (shape, stalk) is similar to those described in *P. isidori* (Kirschbaum 1981) and other mormyrid species (*H. bebe*, *M. rume*, *M. deliciosus*, Kirschbaum 1995). The larval electric organ degenerates (Kirschbaum 1981) after the activation of the adult electric organ. We do not have histological data proving a degeneration in *C. rhynchophorus*; however, our EOD data of pure adult electric organ discharge beyond a certain developmental stage support the conclusion that the larval EO has degenerated throughout ontogeny.

### Adult electric organ

In *C. rhynchophorus*, early during ontogeny there is a transitional stage when the EODs of the larval electric organ and those of the adult electric organ are superimposed. This is similar to data from *Marcusenius macrolepidotus* (Werneyer and Kramer 2006). After this short transitional stage, only the adult EO is active, but its discharge in the juvenile *C. rhynchophorus* differs considerably from that in the adult, both in shape and duration. These changes occur gradually throughout at least a year. This pattern differs from that of *M. macrolepidotus* where the juvenile EOD does not change during ontogeny, but stays constant up to adulthood without any change in shape or duration. The EOD of *M. macrolepidotus* is biphasic with a head-positive phase followed by a head-negative phase of similar amplitude and duration, and is terminated by a very weak head-positive postpotential. The total duration of this EOD amounts to about 700 µs (Werneyer and Kramer 2006).

In principle, physiological and/or anatomical features could contribute to such a considerable ontogenetic change of the EOD in *C. rhynchophorus*. We show that the electrocytes of *C. rhynchophorus* develop during ontogeny surface proliferations on the rostral face (i.e., papillae) which could well be responsible for the increase in the total duration of the EOD, as already discussed by Paul et al. (2015) for *C. numenius* and by Bass et al. (1986) for *Brienomyrus brachyistius* [long biphasic) (later identified as *Paramormyrops kingsleyae* (Hopkins et al. 2007)]. Schwartz et al. (1975) made a calculation concerning the increase of surface area due to papillae: papillae 100 µm in diameter and 150 µm long can lead to a total increase of surface area by a factor of about 110. We have described the papillae using a light microscope. A more detailed fine structural description (EM techniques) of these structures would be useful. Increase of surface area can also be obtained by tubular surface invaginations (Schwartz et al. 1975; Bass et al. 1986). Our light microscopical investigations do not reveal, whether such invaginations are present in *C. rhynchophorus.*

Holes and cavities in electrocytes are more prominent in the older stages of *C. rhynchophorus.* Our ontogenetic sequence suggests that the cavities might later on develop into the holes (Fig. 10e–f). It may be possible, that these holes contribute to some extent to the increase in EOD duration in juveniles longer than 10 cm (Fig. 5). The holes would delay the establishment of a potential across the electrocyte membrane due to a leakage of ions. Holes and irregularities in the electrocyte anatomy (Fig. 8) are different structures which can be deduced from the course of the connective tissue sheath surrounding the individual electrocytes (compare Figs. 8 and 10). We cannot see how the few irregular electrocytes could have an influence on EOD duration or shape.

During ontogeny, the EOD of *C. rhynchophorus* changes from a biphasic into a triphasic EOD, while the basic geometry of the electrocytes does not change: the main stalk is located at the caudal face and penetrations are absent. One explanation could be that the activation of the rostral by the caudal face (Bennett 1971) is pertained for a long time, thus exceeding the duration of the activation of the caudal face. Such a triphasic EOD of about 200 µs duration is, e.g., produced by *P. isidori* (Westby and Kirschbaum 1982), a species which does not possess papillae (Denizot et al. 1982).

In Fig. 4, we show that the inter-individual variation of the EOD both concerning shape and duration is very small. The largest individual variation is seen in the 60- to 64-mm-long fish (Fig. 4d). At this stage, the papillae become more prominent (Fig. 9). These individual variations in EOD may relate to individual differences in the differentiation of the papillae. Regarding potential physiological parameters underlying the ontogenetic change in EOD seen in *C. rhynchophorus*, Nagel et al. (2017) correlated the long EOD of *C. tshokwe* (4 ms as compared to the short EOD of *C. compressirostris* of about 200 µs duration) with a significantly elevated expression of voltage-gated ion channel genes in *C. tshokwe*. The juvenile EODs of both species, however, are nearly identical (Kirschbaum et al. 2016; Nguyen et al. 2017). Therefore, the differential expression apparently is not yet established in the early juvenile of *C. tshokwe*. If a similar scenario would be valid for *C. rhynchophorus*, the ontogenetic change in the shape of the EOD could also be correlated with an ontogenetic increase in the expression of voltage-gated ion channel genes. This hypothesis, however, remains to be tested, which seems well feasible, as *C. rhynchophorus* can be bred quite easily in captivity. It is quite probable that physiological developmental changes also relate to the observed morphological changes of the electrocyte membrane. A potential candidate for the explanation of EOD changes during ontogeny could also be the kcna7a channel gene which is predominantly expressed in mormyrid electric organs (Immani et al. 2018).

In this paper, we describe the whole ontogenetic sequence from the early juvenile up to the adult fish. Interestingly, the EOD typical for the adult was produced by the fish for the first time when they had reached the minimum size for first sexual maturity, i.e., at a length of about 15 cm. This is to be expected, as the adult EOD is used in the context of reproductive behavior and has relevance for reproductive isolation of sympatric closely related species (Feulner et al. 2008; Nagel et al. 2018a, b).

The adult EOD as a tool for species identification is robust in species with no ontogenetic changes in EOD. In species with ontogenetic changes of EOD, as in *C. rhynchophorus* or *C. numenius* (Paul et al. 2015), juvenile EODs erroneously taken as species identification tool can lead to misidentification of species.

## Conclusions

The change in shape and duration of the EOD in *C. rhynchophorus* can to a certain extent be explained by structural changes of the electrocytes during ontogeny. Our light microscopical studies provide a first description of the anatomy of the papillae. Further electron microscopical studies (both transmission and scanning techniques) would be useful to increase our knowledge in this respect, if applied in a comparative way across differentially discharging species/ontogenetic stages in the genus *Campylomormyrus.*

## Electronic supplementary material

Below is the link to the electronic supplementary material.

Supplementary material 1 (R 3 kb)

## Abbreviations

EO: Electric organ
EOD: Electric organ discharge
