# Correction to: Ontogeny of electric organ and electric organ discharge in *Campylomormyrus rhynchophorus* (Teleostei: Mormyridae)

**DOI:** 10.1007/s00359-021-01519-w

**Published:** 2021-11-08

**Authors:** Linh Nguyen, Victor Mamonekene, Marianne Vater, Peter Bartsch, Ralph Tiedemann, Frank Kirschbaum

**Affiliations:** 1 Unit of Evolutionary Biology/Systematic Zoology, Institute of Biochemistry and Biology, University of Potsdam, Karl‑Liebknecht‑Str. 24‑25, Haus 26, 14476 Potsdam‑Golm, Germany; 2 Unit of Biology and Ecology of Fishes, Faculty of Life Sciences, Humboldt University of Berlin, Philippstr. 13, Haus 16, 10115 Berlin, Germany; 3 Ecole Nationale Supérieure d’Agronomie Et de Foresterie, Université Marien Ngouabi, B.P. 69, Brazzaville, Republic of Congo; 4 Unit of General Zoology, Institute of Biochemistry and Biology, University of Potsdam, Karl‑Liebknecht‑Str. 24‑25, Haus 26, 14476 Potsdam‑Golm, Germany; 5 Leibniz‑Institute for Evolution and Biodiversity Science, Museum fuer Naturkunde Berlin, Invalidenstr. 43, 10115 Berlin, Germany

## Correction to: Journal of Comparative Physiology A (2020) 206:453–466 10.1007/s00359-020-01411-z

The article “Ontogeny of electric organ and electric organ discharge in *Campylomormyrus rhynchophorus* (Teleostei: Mormyridae)”, written by Linh Nguyen, Victor Mamonekene, Marianne Vater, Peter Bartsch, Ralph Tiedemann and Frank Kirschbaum, was originally published Online First without Open Access. After publication in volume 206, issue 3, page 453–466 the author decided to opt for Open Choice and to make the article an Open Access publication. Therefore, the copyright of the article has been changed to © The Author(s) 2021 and the article is forthwith distributed under the terms of the Creative Commons Attribution 4.0 International License, which permits use, sharing, adaptation, distribution and reproduction in any medium or format, as long as you give appropriate credit to the original author(s) and the source, provide a link to the Creative Commons licence, and indicate if changes were made. The images or other third party material in this article are included in the article’s Creative Commons licence, unless indicated otherwise in a credit line to the material. If material is not included in the article’s Creative Commons licence and your intended use is not permitted by statutory regulation or exceeds the permitted use, you will need to obtain permission directly from the copyright holder. To view a copy of this licence, visit http://creativecommons.org/licenses/by/4.0. Open Access funding enabled and organized by Projekt DEAL.

The original article has been corrected.

